# Recursive Cluster Elimination Based Support Vector Machine for Disease State Prediction Using Resting State Functional and Effective Brain Connectivity

**DOI:** 10.1371/journal.pone.0014277

**Published:** 2010-12-09

**Authors:** Gopikrishna Deshpande, Zhihao Li, Priya Santhanam, Claire D. Coles, Mary Ellen Lynch, Stephan Hamann, Xiaoping Hu

**Affiliations:** 1 Department of Electrical and Computer Engineering, Auburn University MRI Research Center, Auburn University, Auburn, Alabama, United States of America; 2 Department of Biomedical Engineering, Georgia Institute of Technology and Emory University, Atlanta, Georgia, United States of America; 3 Department of Psychiatry and Behavioral Sciences, Emory University School of Medicine, Atlanta, Georgia, United States of America; 4 Department of Psychology, Emory University, Atlanta, Georgia, United States of America; Indiana University, United States of America

## Abstract

**Background:**

Brain state classification has been accomplished using features such as voxel intensities, derived from functional magnetic resonance imaging (fMRI) data, as inputs to efficient classifiers such as support vector machines (SVM) and is based on the spatial localization model of brain function. With the advent of the connectionist model of brain function, features from brain networks may provide increased discriminatory power for brain state classification.

**Methodology/Principal Findings:**

In this study, we introduce a novel framework where in both functional connectivity (FC) based on instantaneous temporal correlation and effective connectivity (EC) based on causal influence in brain networks are used as features in an SVM classifier. In order to derive those features, we adopt a novel approach recently introduced by us called correlation-purged Granger causality (CPGC) in order to obtain both FC and EC from fMRI data simultaneously without the instantaneous correlation contaminating Granger causality. In addition, statistical learning is accelerated and performance accuracy is enhanced by combining recursive cluster elimination (RCE) algorithm with the SVM classifier. We demonstrate the efficacy of the CPGC-based RCE-SVM approach using a specific instance of brain state classification exemplified by disease state prediction. Accordingly, we show that this approach is capable of predicting with 90.3% accuracy whether any given human subject was prenatally exposed to cocaine or not, even when no significant behavioral differences were found between exposed and healthy subjects.

**Conclusions/Significance:**

The framework adopted in this work is quite general in nature with prenatal cocaine exposure being only an illustrative example of the power of this approach. In any brain state classification approach using neuroimaging data, including the directional connectivity information may prove to be a performance enhancer. When brain state classification is used for disease state prediction, our approach may aid the clinicians in performing more accurate diagnosis of diseases in situations where in non-neuroimaging biomarkers may be unable to perform differential diagnosis with certainty.

## Introduction

Functional magnetic resonance imaging (fMRI) is an effective and non-invasive technology for investigating brain function. Consequently, fMRI data has been extensively used to investigate the neural correlates of healthy and disease states with respect to various sensory, motor and cognitive brain processes. Traditional approaches rely on statistical differences between the data obtained from two different populations or two different conditions within the same population. However, statistical separation of data features between groups does not imply that those features have a predictive value in foretelling the group to which a novel example will belong. Therefore, statistical separation based on hypothesis testing has limited value in the generalizability of the results, a key goal in any scientific endeavor. This has led to the introduction of machine learning approaches into neuroimaging which use a part of the data to learn the rules which discriminate between the groups and which can then be generalized with some accuracy.

Methodologically, most of the neuroimaging studies use a specific machine learning framework for classification. This framework consists of three parts. The first part is pattern analysis for feature extraction where in specific characteristics are obtained from the data with the hope that they will be different for different classes. Commonly used features include voxel intensities [1], [2] and temporal synchrony [3]. The second part is feature selection. Since not all features may distinguish the classes, “filter methods” such as t-tests [4] or “wrapper methods” such as recursive feature elimination (RFE) [3] are employed to select only those features which have the discriminatory power. Generally, the wrapper methods perform better than the filter methods for feature selection [2]. In the third part, the selected features are input to a machine learning algorithm which learns the implicit rule which separates the classes such that it will be able to correctly assign a novel example into the correct class. Both univariate and multivariate methods [2] have been used for classifying neuroimaging data. Multivariate methods based on multiple voxel pattern analysis (MVPA) provide a distinct advantage over the univariate methods [5]. Within MVPA, support vector machines (SVM) have been reported to be very reliable and less sensitive to noise [4].

Strategies using specific combinations of each of the individual parts of the framework described above have been previously used. For example, MVPAs which use the functional connectivity (defined as the instantaneous non-directional temporal correlation between brain regions) information as opposed to just voxel intensities have been shown to perform better [3]. In this regard, SVM based classifiers have been demonstrated to reliably distinguish patients with major depressive disorder (MDD) from healthy controls based on their resting state functional connectivity patterns [3]. Effective connectivity, as opposed to functional connectivity, provides information on the direction of time-delayed causal influences between regions and is expected to improve the classification accuracy. In this regard, another study used multivariate partial least squares and structural equation modeling (SEM) to distinguish MDD patients receiving different treatments based on their effective connectivity patterns derived from SEM [6]. However, since SEM limits the number of regions that can be included in the model and requires *a priori* specification of their connectivity architecture, it has limited utility as a data driven approach and hence does not completely exploit the advantages of SVM, which is fully data driven and which works best when the feature space is adequately sampled.

In order to maximize prediction accuracy, the features extracted from the data must have as much discriminatory power as possible, the feature selection must reliably eliminate, in a computationally efficient manner, the features that do not possess the discriminatory power and the classifier must be able to exploit the discriminatory power available in the selected feature space. While very efficient classifiers have been developed in the machine learning field, their utility in neuroimaging is dependent upon effective feature extraction and selection strategies which are developed in the neuroimaging field. To this end, we propose a novel combination of feature extraction, selection and classification strategies which may maximize prediction accuracy for brain state classification. Our feature extraction is based on a variant of Granger causality (GC), called correlation-purged Granger causality (CPGC), which is capable of inferring the underlying causal brain networks without interference from instantaneous correlation [7], [8]. CPGC can provide the directional causality information without making any *a priori* assumptions about the underlying connectivity architecture or limitations about the number of regions. Owing to the superiority of wrapper methods over filtering methods, we adopt recursive cluster elimination (RCE) [9] for feature selection since it is faster than RFE and also considers feature clusters rather than individual features [9]. Finally, we use a linear SVM classifier as its potency has been amply demonstrated before [1]. This is the first study, to the best of our knowledge, to use effective connectivity for feature extraction in combination with RCE for feature selection for brain state classification.

We illustrate this approach to brain state classification using a specific instance of disease state prediction by successfully predicting whether any given subject was prenatally exposed to cocaine when no significant behavioral differences were found between the two groups. Prenatal cocaine exposure (PCE) can be associated with behavioral problems in children and adolescents that affect occupational, behavioral and emotional functioning [10]–[13]. Such problems may be the effect of alterations in an arousal regulatory mechanism in the brain where one's ability to adjust and allocate mental resources for distinct yet interactive streams of information processing is compromised [14]. Arousal regulation involves multiple brain circuits such as the Default mode network, emotional network and executive control network. Previous neuroimaging task-based studies from our group using fMRI have shown that in subjects prenatally exposed to cocaine, some regions corresponding to the above networks exhibit activity different from those observed in their healthy counterparts [15]. However, other studies which investigated only one of the above networks reported no significant activation differences between the PCE and control groups. For example, Hurt and colleagues found similar activations in the executive control network of both the groups during a working memory task [16]. Given previous studies which have indicated that PCE effects are subtle (despite significant social consequences) [17], finding objective and consistent biomarkers based on neuroimaging data remains a challenge. This makes an interesting test case for the applicability of sophisticated pattern analysis and machine learning approaches for brain state classification.

In this study, we examine the hypothesis that the neurobiological basis of the teratological effects of PCE may involve baseline (or resting state) alterations in the interactions between multiple brain networks and hence may not be apparent in spatially localized task based activations. Given that resting state networks have been shown previously to be sensitive to baseline alterations in various disorders such as cocaine abuse [18], Tourette syndrome [19], multiple sclerosis [20] and Alzheimer's disease [21], we posit that the same would hold true in the case of PCE. In general, resting state also has the advantage of not requiring any task to be performed as it may be difficult for people with clinical conditions to perform certain tasks inside the scanner. Accordingly, we will obtain resting state functional and effective connectivity networks from fMRI data acquired from both healthy and PCE groups and use those as features in our classifier. Finally, we will compare the classifier performance obtained from resting state networks with those obtained from behavioral data, resting state voxel intensities, task activations and task-based networks.

## Materials and Methods

### Subjects

Participants were adolescents recruited from cohorts identified originally as part of two longitudinal studies of PCE on infant development [22], [23]. Both cohorts were drawn from a low income, predominantly African-American population with infants delivered at an urban hospital during 1987–1994. The PCE and control participants in the present study respectively comprised 30 (19M11F, 15.3±2.1 y.o.) and 26 (10M16F, 14.9±2.3 y.o.) participants. The participants used in the present study largely overlapped with those used in our previous study [15]. However, the present study used resting data while the previous study focused on activations; therefore some subjects with good resting data failed to follow task instructions (thus no activation data) and some subjects with good activation data did not have the imaging slice covering the amygdala. Consequently, there was some mismatch between the present sample and that reported previously. The index of sample overlap (ISO) is often used to assess the overlap between samples and is given by
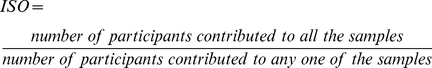



For the two samples used in this paper and our previous paper [15], the ISO is 0.88. Prenatal cocaine exposure was determined by maternal self-report and/or positive urine screen at recruitment post-partum. Positive maternal urine screens at labor and delivery and during pregnancy noted in the medical record were also accepted as evidence of use. More information regarding the determination of substance use, participants' inclusionary criteria, and classification of participants into experimental groups have been described extensively in previous reports [22], [23].

### Data Acquisition and Pre-processing

#### Experimental design

Behavioral data used in the present study were acquired from a cognitive task that the participants performed in separate fMRI activation scans. To investigate interactions between neural responses of working memory and emotion processing, this task had 4 conditions: 0-back task with neutral emotional distraction, 1-back task with neutral distraction, 0-back task with negative distraction, and 1-back task with negative distraction. Participants in this task were instructed to focus on the memory tasks (0- or 1-back) and ignore the emotional distractions. For the memory task, each subject provided 8 behavioral measurements with one accuracy index and one reaction time (only correct responses) for each of the 4 conditions. The accuracy index is the product of the true hit fraction with (1- false alarm fraction) and hence combines both sensitivity and specificity factors. Details about the task design and statistical group comparisons of behavioral performances were reported in our previous publication [15]. Briefly, the memory performance decreased with either higher memory load or/and negative emotional distraction. However, as the task paradigm was deliberately designed to minimize behavioral group difference, no significant group differences in task performance were observed.

#### MRI data

With a 3T MRI scanner (Siemens Medical Solutions, Malvern, PA), both the resting-state and task scans used a T_2_*-weighted echo-planar imaging sequence. The acquisition parameters for the task were: 120 volumes per scan, matrix = 64×64, 30 axial slices, 3 mm in thickness without gap, repetition time (TR)/echo time (TE) = 3000 ms/30 ms, flip angle = 90°, field of view (FOV) = 192 cm. For the resting state scan, the parameters were: 210 time points, matrix = 64×64, 20 axial slices without gap, slice thickness = 4 mm, TR/TE = 2000 ms/30 ms, flip angle = 90°, FOV = 192 cm. Corresponding high resolution (256×256) 3D T_1_-weighted anatomical images were also acquired for each subject. Image preprocessing for the task data followed the standard pre-processing pipeline while that for the resting state data included slice timing correction, rigid body registration, regressing out of white matter and CSF time series and 0.009 Hz<f<0.08 Hz temporal band-pass filtering.

### Regions of Interest (ROI) Selection and Network Identification

Results of our previous fMRI studies [15], [24] showed that PCE could alter brain activation in regions associated with arousal regulation (amygdala and default mode network) and that these alterations in turn affected brain activations involved in cognitive processes (e.g. lateral prefrontal cortex). Based on these previous findings, 9 regions of interest were defined in the present study including bilateral amygdala, bilateral lateral prefrontal cortex (PFC), bilateral parietal cortex, medial prefrontal cortex (MPFC), anterior cingulate cortex (ACC) and posterior cingulate cortex (PCC). The amygdala and cingulate ROIs (PCC and ACC) represented nodes of the emotional network and default mode network, respectively, which together constitute the arousal regulation network, while the PFC and parietal ROIs represented nodes of the executive control network. As the participants performed a working task with emotional distractions, these ROIs were derived from the results (voxel-wise p<0.001, uncorrected) of a 2 (high vs. low memory load) ×2 (negative vs. neutral emotional arousal) repeated ANOVA. The bilateral amygdala exhibited the positive emotion effect (BOLD signal higher in the negative condition than neutral); the bilateral prefrontal, parietal and medial prefrontal cortices exhibited the positive memory effect (BOLD signal higher in the 1-back condition than 0-back); and the anterior/posterior cingulate cortices exhibited the negative memory effect (BOLD signal higher in the 0-back condition than 1-back). To avoid biasing the ROIs to activations/deactivations of either group, the ANOVA used equal numbers of participants from both group (23 PCE +23 control subjects). Talairach co-ordinates [25] and volumes of those ROIs are shown in Table 1. Nine time series, each being the average from all the voxels within an ROI, were extracted for subsequent analysis.

**Table 1:** The regions of interest defined from task activations.

Regions of interests	Talairach coordinates*	Volume (mm^3^)
Left amygdala	23.6, 6.9, −10.5	2147
Right amygdala	−25.2, 6.6, −10.7	2481
Left lateral prefrontal cortex	41.5, −7.5, 32.5	2466
Right lateral prefrontal cortex	−41.5, −10.9, 31.5	2294
Left parietal cortex	34.0, 49.5, 42.4	4529
Right parietal cortex	−34.8, 47.6, 44.1	5265
Anterior cingulate cortex	0.6, −48.9, 10.0	12464
Posterior cingulate cortex	2.5, 49.4, 24.3	14090
Medial prefrontal cortex	0.7, −12.3, 46.6	5063

*Coordinates reported in AFNI format (http://afni.nimh.nih.gov/afni/doc/faq/59).

### Correlation-purged Granger Causality

Given *k* time series *X(t)* = [*x_1_(t) x_2_(t)* … *x_k_(t)*], with *k* being 9 in this study, the traditional vector autoregressive (VAR) model of order *p* is given by:
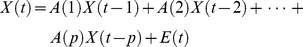
(1)where *E(t)* is the model error and *A(1) … A(p)* are the coefficients of the VAR model. Multivariate Granger causality can be derived based on the model coefficients *A(1) … A(p)* as in previous studies [26]–[30].
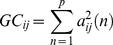
(2)where *a_ij_* are the elements of the matrix *A*. We introduced the zero-lag term into Eq.1 as shown below to account for the zero-lag correlation effects.
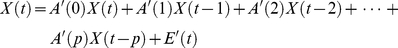
(3)


Eq.3 represents a modified VAR (mVAR) model where the diagonal elements of *A'(0)* are zero such that only the instantaneous cross-correlation, and not the auto-correlation, between the time series are modeled. The model coefficients obtained from Eq.3 are not equal to those obtained from Eq.1, i.e. *A'(1) … A' (p)*≠*A(1) … A(p)* because the inclusion of the zero-lag term affects the value of other coefficients. GC obtained from *A'(1) … A' (p)* are free from the effects of zero-lag correlation and is defined as correlation-purged GC (CPGC) [7], [8].
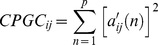
(4)


In addition, the zero-lag correlation between the time series is given by *A'(0)*. The mVAR model's order was determined using Bayesian Information Criterion (BIC) [31].

### Feature Extraction

Different types of features were derived from both task-based and resting state fMRI data and behavioral data as described below.

1. *Behavioral data*: The accuracy index and reaction time obtained from each of the 4 experimental conditions.
2. *Task Activation*: The beta values obtained from the activation GLM for each of the 9 ROIs and for each of the 4 conditions.
3. *Resting State Voxel Intensities*: Classifiers dealing with task-based fMRI data usually consider the beta values at each voxel or time series from activated voxels obtained from a general linear model as input features [2]. However, this approach cannot be utilized in the absence of experimental modulation of brain activity such as during resting state. Consequently, the entire resting state time series of each ROI was selected as multidimensional input features to the classifier. This enabled us to compare the efficacy of univariate features as opposed to connectivity features which are multivariate in nature.
4. *Resting State Connectivity*: The time series representing the 9 ROIs for each subject was input into the mVAR model described before, to obtain both causal and instantaneous correlation networks for every subject. First, the path weights of the instantaneous correlation networks were input into the RCE-SVM classifier. Subsequently, both causal and instantaneous connectivity features were used as inputs. Finally, instantaneous connectivity was obtained from the traditionally used Pearson's correlation and used as features in the classifier for comparison. In addition, a t-test was performed to identify the connectivity features which were significantly different between the two groups.
5. *Task-based connectivity*: The procedure described in the previous paragraph was applied to task-based data instead of resting state data.

### Recursive Cluster Elimination based Support Vector Machine (RCE-SVM) Classifier

SVM is a machine learning approach developed by Vapnik [32] and has been extensively used for classification in many different fields [33]. It has been previously demonstrated that using discriminatory features, i.e. those features which assume statistically different values for the classes under consideration, enhances the performance of SVM-based classification [3]. To this end, filtering methods and wrapper methods have been used [3]. The filtering approach is based on using statistical tests such as t-test to select features which are statistically different between the classes. Wrapper methods such as RFE and RCE are based on iteratively eliminating features so as to minimize the prediction error. In wrapper methods such as RCE, the feature selection/elimination and classification steps are embedded with each other and are repeated after each iteration. Therefore, we refer to it as the RCE-SVM classifier. The potency of the RCE-SVM classifier has been previously established in the context of gene classification [9], though this is its first application to the field of neuroimaging to the best of our knowledge.

**Figure 1:**
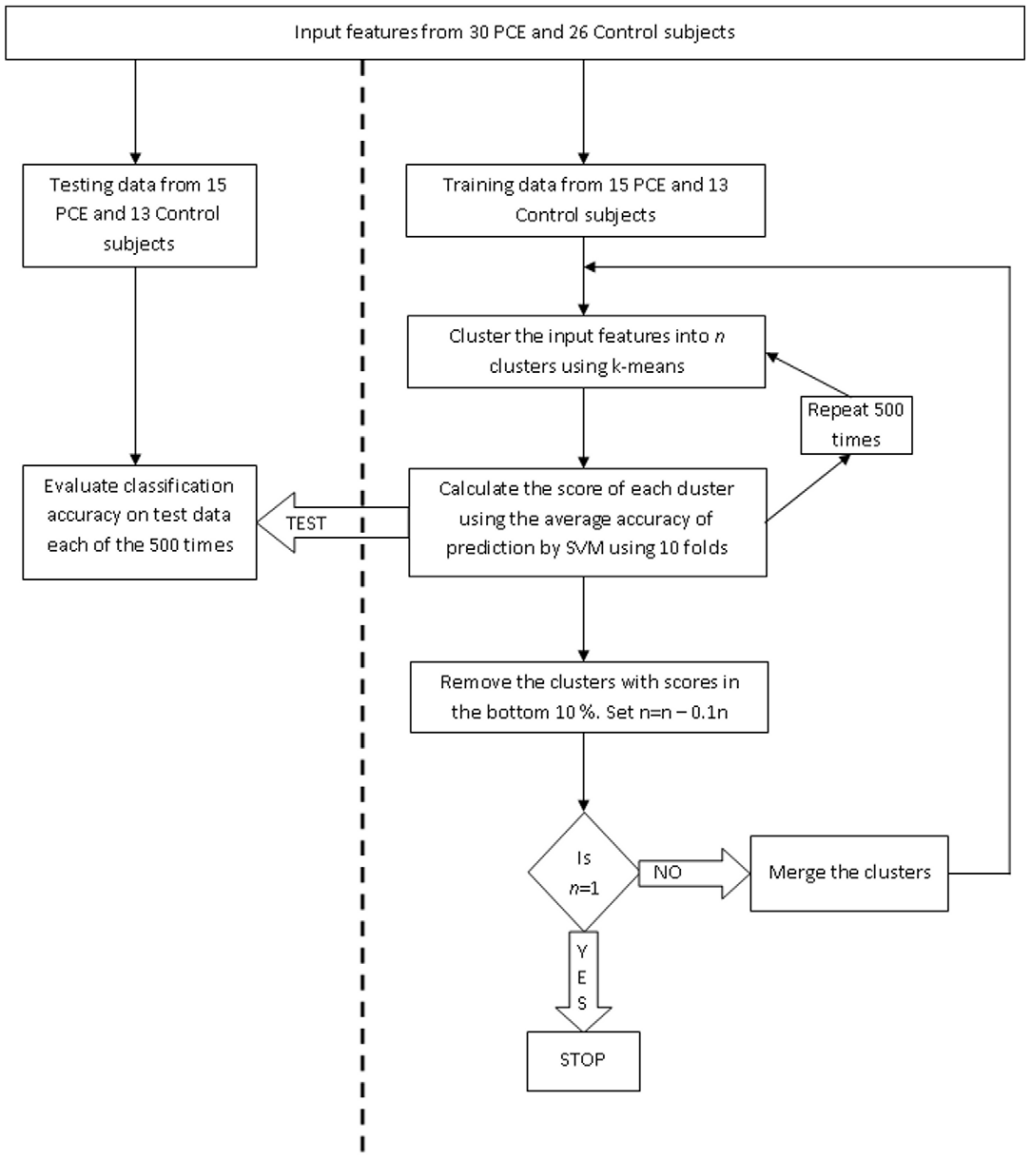
Flow chart depicting the RCE-SVM procedure.

The main steps of the RCE-SVM algorithm, shown in the flowchart in Fig. 1, are the cluster step, the SVM scoring step and the RCE step. First, the input features for all the 56 subjects (30 PCE and 26 healthy subjects) were partitioned into two parts, each containing 15 PCE and 13 control subjects. The first part was used for training and the second part for testing. In the clustering step, the training data was clustered into *n* clusters using *K*-means algorithm [34]. The number of clusters was first set to the number of features and was progressively decreased by one until there were no empty clusters. The *n* obtained by this iteration served as the initial *n* for the RCE-SVM loop. In the SVM scoring step, the score of each cluster, defined as its ability to differentiate the two classes of samples by applying linear SVM, was obtained. In order to calculate the score of each cluster, we randomly partitioned the training data into 10 non-overlapping subsets of equal sizes (10 folds). Linear SVM was trained using 9 subsets and the remaining subset was used to calculate the performance. The clustering and cross-validation procedure was repeated 500 times in order to take into account different possible partitionings. The average accuracy of the SVM over all the folds and repetitions was designated as the score of the corresponding cluster. For each of the 500 repetitions, classification accuracy was ascertained using the test data. In the RCE step, the bottom 10% of the clusters with the lowest score was eliminated. The surviving features were merged, *n* was decreased by 10% and the above three steps performed again in an iterative fashion. With every successive iteration, the testing data was used to assess the performance of the classifier with a lower number of features compared to the previous iteration. The complete separation of training and testing data also ensures that there is no bias in the performance accuracy [35]. The procedure was terminated when the number of clusters was equal to one. However, the classifier performance was plotted only until maximum accuracy with minimum number of features was obtained. The features corresponding to that iteration were tabulated and rank ordered based on their scores. The evolving accuracy was calculated at every RCE-SVM loop as the mean accuracy of all 500 repetitions calculated at each loop using the feature clusters of test data available at the corresponding loop (Fig. 1).

## Results

**Figure 2:**
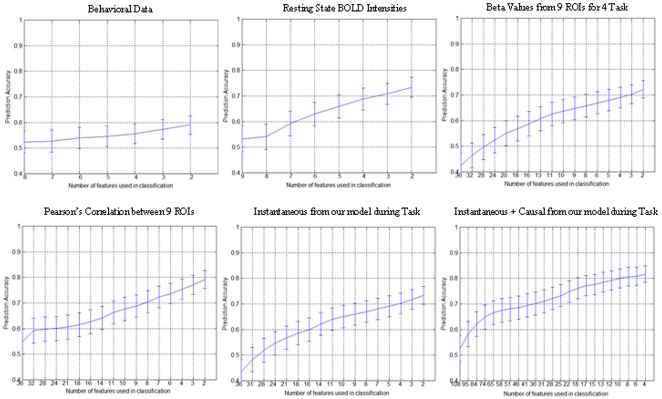
The evolving performance of the RCE-SVM classifier with decreasing number of features derived from: top left- behavioral data obtained from a working memory task with emotional distracters, top middle- resting state BOLD intensities from 9 ROIs, top right- beta values from 9 ROIs for 4 task activation conditions, bottom left- Pearson's correlation between 9 ROIs during task, bottom middle- instantaneous influence from our model during task, bottom right- instantaneous + causal influence from our model during task.

The evolving accuracy of the RCE-SVM classifier using features from behavioral data, resting state BOLD intensities and task activations is shown in the top panel of Fig. 2. Table 2 shows the corresponding maximum accuracies achieved and the ranking of the features for maximum accuracy based on their SVM scores. It is evident that the final accuracy of 59% obtained from behavioral data is just above chance and of no practical value. Among the behavioral features, the ones for the negative 0-back condition seemed to carry limited discriminatory information between the groups. As reported before in our previous study [15], 2 (emotion effect, neutral vs. negative) ×2 (memory effect, 0-back vs. 1-back) ×2 (exposure, PCE vs. control) ANOVA revealed significant emotion, memory and emotion × memory interaction effects, but no group differences between PCE and controls. Even though the performance of the classifier using resting state voxel intensities and task beta values was better than that using behavioral data, the final accuracies of 73.4% and 72.3%, respectively, is not high enough for use in practical applications. Resting state voxel intensities from PCC and R Parietal and task beta values from R Parietal negative 0-back condition and L PFC Negative 1-back condition provided maximum accuracy, respectively.

**Table 2:** Maximum accuracy and important features for different metrics.

Metric	Maximum % Accuracy	Features providing maximum accuracy	Rank
Behavioral Data	59	Reaction Time Negative 0-back	1
		Accuracy Index Negative 0-back	2
Resting State BOLD intensities from 9 ROIs	73.4	Posterior Cingulate	1
		Right Parietal	2
Beta Values from 9 ROIs for 4 Task Conditions	72.3	Right Parietal Negative 0-back	1
		Left PFC Negative 1-back	2

	Pearson's Correlation	Instantaneous Influence from our model	Instantaneous + Causal Influence from our model	Features providing max accuracy	p-value	Rank
Resting state connectivity weights with CSF/WM regression	84	80.1	90.3	L Amygdala →R PFC	0.001 (PCE>control)	1
				R Amygdala →R PFC	0.003 (PCE>control)	2
				MPFC →R PFC	0.03 (PCE>control)	3
Resting state connectivity weights without CSF/WM regression	80.1	78.3	88.6			
Connectivity weights during task	79.2	73.3	81.7	PCC → R Amygdala	0.04 (control>PCE)	1
				ACC → PCC	>0.05	2
				R PFC → PCC	0.03 (control>PCE)	3
				L Parietal → L Amygdala	0.04 (control>PCE)	4

The evolving accuracy of the RCE-SVM classifier using task-based connectivity features is shown in the bottom panel of Fig. 2. It can be seen that as the RCE algorithm eliminated the features, the accuracy of classification steadily improved up to 79.2%, 73.3% and 81.7% for Pearson's correlation, instantaneous influence from our model and instantaneous + causal influence from our model, respectively. Frontal/Cingulate/Parietal → Amygdala top-down causal paths, i.e. PCC → R Amygdala and L Parietal → L Amygdala with ACC and R PFC feeding into PCC (Table 2), were the features providing maximum accuracy with task-based connectivity. In addition, 3 of the 4 paths, i.e. PCC → R Amygdala, R PFC → PCC and L Parietal → L Amygdala, were significantly higher (p<0.05) in controls as compared to PCE group.

**Figure 3:**
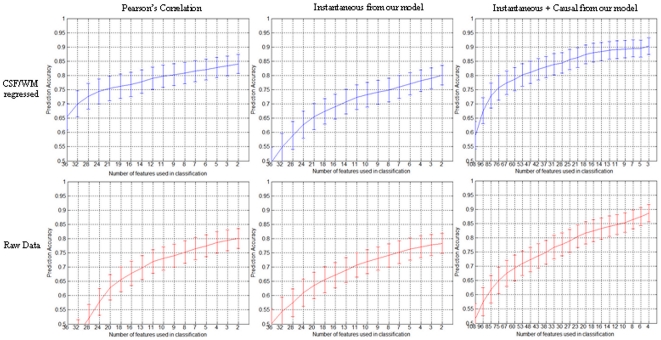
The evolving performance of the RCE-SVM classifier with decreasing number of features derived from: top row – data regressed with CSF and white matter time series, bottom row – data not regressed with CSF/WM time series, left column – Pearson's correlation from resting data, middle column – instantaneous influence from our model from resting data, right column – instantaneous and causal influence from our model from resting data.

The evolving accuracy of the RCE-SVM classifier using resting state connectivity features obtained from data regressed with CSF and white matter (WM) time series is shown in the top panel of Fig. 3, while the curves for data without regression are shown in the bottom panel of Fig. 3. Table 2 shows the corresponding maximum accuracies achieved and the ranking of the features for maximum accuracy based on their SVM scores. For all the three connectivity feature sets, i.e. Pearson's correlation, instantaneous influence from our model and instantaneous + causal influence from our model, regressing out the CSF and WM time series from resting state data improved the final accuracy from 2–4%. The improvement seems to be larger with Pearson's correlation than with instantaneous and causal influences from our model. The maximum accuracy of 90.3% was achieved by causal paths L Amygdala → R PFC, R Amygdala → R PFC and MPFC → R PFC obtained from data with CSF/WM regression. In addition, all the three paths had a significantly (p<0.05) higher causal influence in PCE group as compared to controls.

## Discussion

### RCE-SVM Classifier Performance

Figs. 2 and 3 demonstrate that without recursive cluster elimination, the performance of the SVM classifier using all the available features would have been 52.3%, 53.2%, 42.5%, 52.5% and 59% for behavioral data, resting state voxel intensities, beta values from task activations, task-based instantaneous plus causal connectivity and resting state instantaneous plus causal connectivity features from CSF/WM regressed data, respectively. In comparison, the corresponding accuracies obtained with RCE-SVM were 59%, 73.4%, 72.3%, 81.7% and 90.3%, respectively. This demonstrates that feature extraction and feature selection are central to the utility of machine learning for brain state classification. Specifically, resting state effective connectivity features seem to provide an edge over other characterizations of brain state for the following reasons. First, as in the case of PCE, different brain states may not necessarily give rise to different behavior and using behavioral data for brain state classification may not inform us about the neural correlates of behavior. Second, in situations where disease states are better characterized by baseline alterations in the brain, rather than during engaging specific brain systems during task performance, univariate features based on voxel intensities or activation beta values may have only limited discriminatory capability. Third, many brain states in healthy and disease populations are characterized by different modes of interaction between brain regions rather than activity within a given region. In such situations, *connectivity*, rather than *activity*, is likely to be discriminatory between different brain states. Accordingly, we observed improved accuracy with task-based connectivity measures as compared to task-based activation measures. However, connectivity measures further improved the performance when they were derived from resting state data rather than task-based data. This corroborates previous studies which have shown that correlation-based functional connectivity metrics from resting state data may provide useful information about temporal synchrony of different regions which may aid in classification [3], [36]. However, since functional connectivity lacks directionality information, it may not always discriminate between brain states if the underlying neural correlates for discrimination depend on the pattern of causal influences between brain regions. As we have demonstrated in the case of PCE, the causal information seems important for obtaining high accuracy.

It is worth noting that removing physiological confounds from resting state data using methods such as CSF/WM regression seems to be useful because it purges the data of non-discriminatory artifacts and hence provides higher accuracy (Table 2). Any other method for removing physiological artifacts in the image [37] or frequency [38] domains must have a similar effect. The comparative performance of Pearson's correlation, instantaneous and causal influence from our model shows that Pearson's correlation performs better than the instantaneous influence from our model while the causal influences from our model outperforms both the instantaneous metrics. Owing to the smoothing of neuronal activity by the hemodynamic response, relatively rapid neuronal influences occurring at time scales finer than the sampling interval may contribute to Pearson's correlation between time series. However, the mVAR model removes those causal influences from the instantaneous term in the model and it is instead reflected in the causal terms. Consequently, the instantaneous term from the mVAR model taken alone may be less informative than Pearson's correlation. Instead, including both the instantaneous and causal terms from our model will be more powerful than Pearson's correlation because it conveys purer estimates of both types of interactions.

The relative merits of wrapper and filter methods for feature selection in fMRI are debatable. While de Martino *et al* [2] found better performance with RFE, the study by Craddock *et al* [3] found that filter method performed better. However, we believe that evidence from the use of these methods in other fields [9] clearly points towards the superiority of wrapper methods and hence we have employed it in this study.

Previous studies have indicated that network metrics could be an important discriminant while studying disorders which alter the topology of networks [36], such as in depression and schizophrenia, where in the small-worldness of the network is compromised [39]. However, in the case of PCE, the hypothesis is that the relative direction and strength of the influence between the sub-cortical structures involved in emotion and the fronto-parietal structures are different in controls as compared to PCE subjects. Hence, we did not investigate network-level metrics such as average number of links or in-out count per node. Future applications of this method to other disorders such as depression will investigate this aspect.

In the literature, many researchers have suggested the usage of nonlinear SVMs so as to obtain a maximum-margin hyper plane in a transformed feature space when the linear classifier is not able to obtain high accuracy in the original feature space [32], [40]. This approach is appropriate when the discriminatory power of the input feature space could no longer be improved using pattern analysis. In our case, we have shown that this is not the case as demonstrated by increased discriminatory power of features derived from brain networks as compared to more traditional features derived from behavioral data or voxel intensities. In addition, the computational burden imposed by nonlinear SVMs may hinder the practical application of this approach in the clinic, where in quick decision making is important. Therefore, we have persisted with linear SVMs in this work.

### Scientific Significance of the Results to PCE

The precise way in which PCE related structural and functional brain changes cause cognitive or behavioral deficits are far from clear. Since not all PCE children suffer from neurobehavioral problems, it is hypothesized that the brain has compensatory mechanisms to provide relief from the effects of PCE. Consequently, general IQ and neurobehavioral tests used to assess children who were prenatally exposed to cocaine may not be sensitive to the factors that are altered in PCE [41]. From the clinical point of view, research that enables healthcare providers to identify biological markers in children at risk, are definitely needed. The present study, employing a pattern analysis and machine learning approach based on resting-state fMRI and effective connectivity, is a step in this direction. Besides the high accuracy in group classification, results shown in the group differences, in terms of features with the most discriminatory power, may be informative about the neurobiological basis of arousal dysregulation reported in previous behavioral and neuroimaging studies of PCE [14], [15], [24], [42]. During resting state, higher effective connectivity from bilateral amygdala to R PFC was observed in the PCE group. Since amygdala is typically involved in processes involving emotion and arousal regulation [43], the higher amygdala to PFC influence may suggest that the exposed adolescents may have a higher baseline arousal level, which in turn affects neural activity in the executive control network [15]. The medial prefrontal cortex is generally considered to be involved in continuous action monitoring and triggering compensatory adjustments in cognitive control [44]. With a higher emotional distraction from amygdala to PFC, PCE subjects may also need to increase this cognitive monitoring, resulting in increased effective connectivity from MPFC to R PFC. While performing the task, our previous study had shown that the PCE subjects could not suppress their amygdala activation with increased memory load [15]. This generally indicates reduced top-down inhibition from prefrontal and parietal regions to amygdala. In this study, we observed lower causal influence from PCC/Parietal regions to bilateral amygdala in PCE subjects as compared to controls and this feature provided maximum accuracy in distinguishing the groups based on task-based effective connectivity, thus supporting our previous results. In addition, the lower R PFC to PCC influence in the PCE subjects suggests that the exposure reduced the inhibitory effect from the executive network to default mode network, which may also underlie arousal dysregulation observed in exposed adolescents [24].

### General Significance of the Results

The framework adopted in this work is quite general in nature with PCE being only an illustrative example of the power of this approach. While it is difficult to claim that the conclusions of this paper will generalize broadly to other data sets, our preliminary investigation of the applicability of this method to other disorders such as depression has yielded positive results [45]. Brain state classification using neuroimaging data has applications in brain-computer interfaces [46], [47], lie detection [48], emotion detection [49] and neurofeedback systems [50], where including the directional connectivity information may prove to be a performance enhancer. When brain state classification is used for disease state prediction, our approach may aid the clinicians in performing more accurate diagnosis of diseases in situations where non-neuroimaging biomarkers may be unable to perform differential diagnosis with certainty.

### Conclusions

In this study, we have introduced a novel framework for brain state classification using both instantaneous and causal resting state connectivity derived from CPGC analysis of fMRI data as features, in conjunction with RCE-based feature selection and SVM-based classification and compared its performance to classification using behavioral metrics, voxel intensities, task activations and task-based connectivity as features. We have demonstrated the efficacy of the CPGC-based RCE-SVM approach using a specific instance of brain state classification exemplified by disease state prediction. We were able to predict, with 90.3% accuracy, whether any given human subject was prenatally exposed to cocaine or not, even when no significant behavioral differences were found between exposed and healthy subjects. This study provides a template, which can be extended to any brain state classification problem, especially those trying to exploit baseline differences in brain function.
